# Endo-esthetic Management of Permanent Maxillary Lateral Incisor with Severe Root Dilaceration in a 12-year-old Child

**DOI:** 10.5005/jp-journals-10005-1006

**Published:** 2008-12-26

**Authors:** Vishwas Chaugule, Chetan Bhat, Vishwas Patil, Krupali Shah, Shirish Vihapure

**Affiliations:** 1 Professor, Head and PG Guide, Department of Pediatric and Preventive Dentistry, Dr DY Patil Dental College and Hospital, Pimpri, Pune, India; 2 Lecturer, Department of Pediatric and Preventive Dentistry, Dr DY Patil Dental College and Hospital, Pimpri, Pune, India; 3 Third year PG student, Department of Pediatric and Preventive Dentistry, Dr DY Patil Dental College and Hospital, Pimpri, Pune, India; 4 Second year PG student, Department of Pediatric and Preventive Dentistry, Dr DY Patil Dental College and Hospital, Pimpri, Pune, India; 5 First year PG Student, Department of Pediatric and Preventive Dentistry, Dr DY Patil Dental College and Hospital, Pimpri, Pune, India

**Keywords:** Root dilacerations, Endo-esthetic management, Fiber post and core buildup.

## Abstract

Functional, esthetic and endodontic restoration of a pulpally involved permanent incisor with root dilaceration often presents a daunting clinical challenge. The outcome of conventional treatment modalities like surgical removal of the tooth followed by orthodontic closure of the space is time consuming and esthetically compromising. Even the prosthetic and implantalogical rehabilitation after extraction is not possible until the patient reaches certain age; while the compliance is a problem with the use of removable partial denture in young children. Autoalloplastic anterior tooth transplantation can lead to physical and psychological trauma in a young individual.

Thus endo-esthetic management of such teeth helps in maintaining both morphology and esthetics in a growing child until the permanent long lasting prosthetic solution is sought after the complete development of the dentition and jaws.

This treatment option for a pulpally involved permanent incisor with root dilaceration involves completing the endodontic treatment in a partially calcified and aberrantly located root canal followed by the use of light transmitting fiber post and core build up using composite resin.

## INTRODUCTION

One of the sequelae to trauma to the primary dentition is the possibility of damaging permanent successor tooth buds. 1 A 69% incidence of developmental disorders to permanent teeth following trauma to the primary teeth has been cited in the literature.^2^

Intrusive displacement of deciduous teeth is the most frequent cause of sequelae involving teeth of the second dentition, with damage ranging from coronal changes in color and shape to radicular malformation and distinct root anomalies. 1,3

The creasing or bending of the crown axis with respect to the root axis, as commonly observed in these cases, is also termed dilaceration. This results from the displacement of the crowns of the incisors, usually still without roots, in a vestibular direction while root growth is still progressing in a cranial direction^4^

The incidence of root dilacerations amounts to approximately 3% of all the damage to permanent teeth following trauma to deciduous teeth. In the literature a distinction is made between vestibular and lateral root bending. Whether the trauma is the sole cause of vestibular root bending is a subject of controversy. In the majority of the cases it has been observed only in the upper central incisor. In many cases the roots are by then malformed and root growth has come to an end prematurely. ^1,3,5- 7^

When such dilacerated teeth gets involved pulpally due to caries, it poses a challenging endo-esthetic problem.

## CASE REPORT

A 12-year-old healthy male child with no contributory medical history visited the Dept. of Pediatric and Preventive Dentistry, Dr DY Patil Dental College Pune, with the chief complaint of pain in the upper front tooth since 15 days and history of intermittent dull ache in upper right and left back region for a couple of months.

**Fig. 1:**
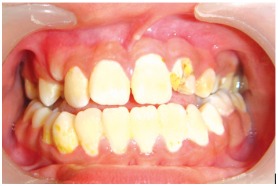
Labial view of the involved tooth

**Fig. 2:**
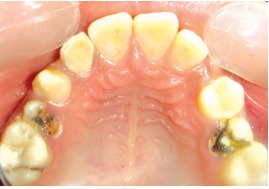
Palatal view of the involved tooth

**Fig. 3:**
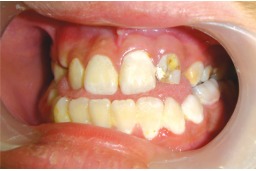
Occlusion

On examination, the upper left lateral incisor showed a gross carious involvement of crown. Upper right and left second premolars showed severe carious destruction of crown rendering them clinically nonrestorable (Figs 1 and 2). The upper left lateral incisor was tender on vertical percussion. The child had mandibular prognathism and angles class III molar relation ship (Fig. 3).

**Fig. 4:**
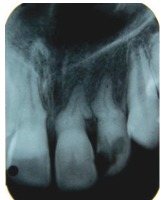
Preoperative radiograph of the involved tooth

Radiographic examination of the upper anterior region revealed tortuous root canals suggestive of severe root dilacerations of upper left central and lateral incisor. In case of lateral incisor the root tortuousity does not seem to be as complicated as of a central incisor. This is because of the buccopalatal angulation of the root of lateral incisor which is not evident on radiograph very clearly (Fig. 4).

Patient’s parents revealed history of traumatic episode at the age four which involved avulsion of two deciduous teeth from upper front region.

 Electric pulp testing of the involved lateral incisor exhibited delayed response compared to the contralateral tooth.

Various treatment modalities were explained to the parents of the child with their pros and cons and it was finally decided to go ahead with the present treatment approach.

**Figs 5 A and B:**
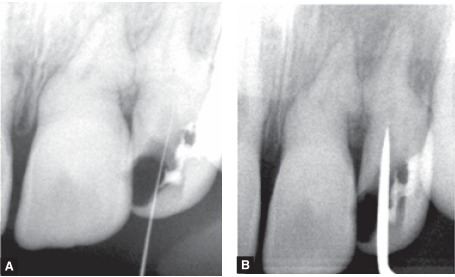
Negotiation of the root canal

As the pulp chamber was calcified and root canal was patent only in the middle and apical third, the negotiation of the root canal was not an easy task (Figs 5 A and B).

Profound anesthesia was achieved by administering 2% Lignocaine with Adrenaline (1:200000) and access cavity preparation was done using round carbide bur.

In an attempt to gain an access to radicular portion, a perforation occurred in coronal one third of the distal aspect of root.

**Figs 6 A and B:**
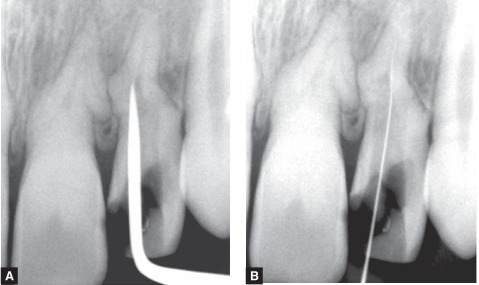
Location of the root canal

Upon further exploration, a very unusual location of the root canal was found on the palatal wall of the root (Figs 6 A and B).

**Figs 7:**
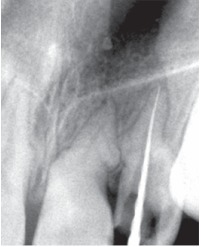
Working length radiograph

An immediate diagnostic radiograph was made to ensure the correct positioning and the length of the root canal (Fig. 7).

**Figs 8:**
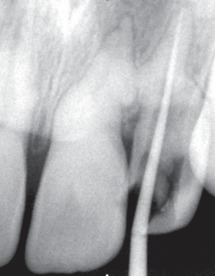
Master cone radiograph

**Figs 9:**
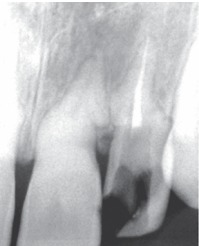
Post-obturation radiograph of the involved tooth

Upon confirming the canal position, thorough biomechanical preparation was carried out using rotary protaper system and pulp space was obturated with gutta percha points using AH-26 (Dentsply, USA) as a root canal sealer (Figs 8 and 9).

**Figs 10:**
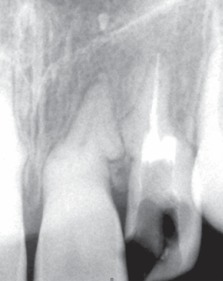
Sealing of perforation with MTA

The perforation on coronal one third of the distal aspect of root was sealed using mineral trioxide aggregate (MTA - Angelus, Brazil) (Fig. 10).

The affected tooth was kept isolated using cotton rolls, 2 × 2 gauze and high vacuum suction all throughout the procedure.

**Figs 11:**
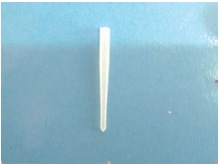
Fiber post

The entire endodontic treatment and sealing of the perforation was carried out in a single visit. In the subsequent visit the post space was prepared using peeso reamers. Considering the length of the root canal and crown-root ratio and the disadvantages associated with cast posts, the light transmitting fiber post (Angelus, Brazil) was used (Fig. 11).

**Figs 12 A and B:**
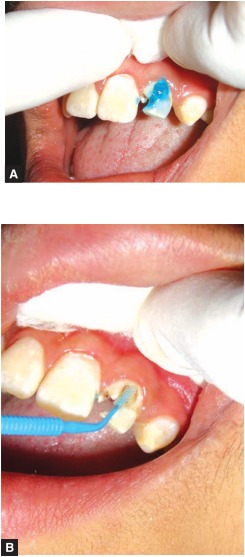
Fiber post cementation procedure

It was cemented using dual cure adhesive cement (Panavia F 2.0 Kuraray Dental products Japan) (Figs 12 A and B).

**Figs 13 A and B:**
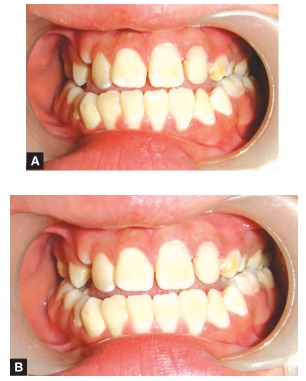
Front view after core build up

The core structure was built around the post using nano filled composite resin (Z-350 3M ESPE USA) (Figs 13 A and B).

**Figs 14 A and B:**
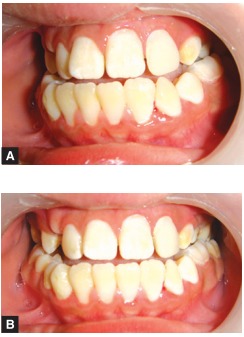
Finishing and polishing

**Figs 15:**
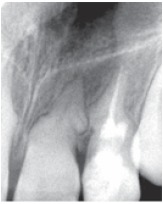
Postoperative radiograph

**Figs 16:**
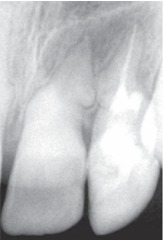
Radiograph at 6-month recall

Finishing and polishing of the composite was done using contouring and polishing discs (Sof-Lex-3M ESPE USA) which ultimately resulted into a fairly acceptable esthetics (Figs 14 to 16).

Considering the dental and chronological age of the child the decision of giving a final extracoronal ceramic restoration is deferred until the eruption of all permanent teeth.

## DISCUSSION

Trauma to primary incisors may lead to lasting damage to the permanent teeth. The patient’s age, and the degree and the direction of the malposition of the primary teeth are some of the factors.

**Figs 17:**
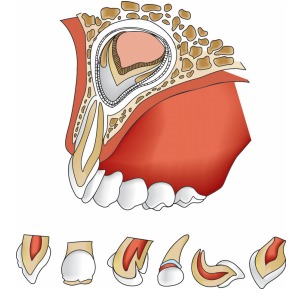
Sequelae to permanent teeth following trauma to primary teeth

Possible sequelae to permanent teeth following trauma involving primary teeth include 8(Fig. 17).

1. Discoloration and hypoplasia of the enamel
2. Bending and malformation of the anatomic crown and root
3. Hypoplasia of the root
4. Retarted eruption.

The maximum incidence of accidents to primary teeth occurs in children aged 3 to 4 years. 9 The crowns of the permanent anterior teeth are already mineralized at this time, whereas root growth is only at the initial stage.^3^

For this reason sequelae to accidents occurring at this time particularly affect root growth. Even in the presented case the parents gave history of trauma at the age of 4 years and avulsion of two primary maxillary anterior teeth.

In such cases various treatment options that would be thought of are surgical removal of the tooth. 5 10Subsequently the preferred therapy is to employ orthodontic methods to close the space or keep the space between the anterior teeth open until the patient reaches an age when definitive implantological or prosthetic treatment can be used. Orthodontic closure of the space is not always indicated nor esthetically satisfactory. Wearing a removable dental prosthesis which has to be regularly adjusted to progressive jaw growth over a period of years may be unsatisfactory for logopedic and psychological reasons. An alternative treatment therefore, in the event of tooth dilaceration can be autoalloplastic anterior tooth transplantation . 10

Just as the above mentioned treatment options have drawbacks, the autoalloplastic transplantation also does inherit disadvantage of a surgical approach in a young individual.

Even an extraction of a tooth with carious crown and a dilacerated root, would pose a traumatic problem.

Considering the drawbacks associated with above mentioned approaches we thought of endo-esthetic management approach with following advantages,

1. Less time consuming
2. Least traumatic physically and psychologically to a child
3. More esthetic
4. Very much predictable in a young child
5. More economical when compared to other treatment modalities.

It remains to be seen the extent of the longevity of the restoration prior to the final permanent restoration in the form of a ceramic extracoronal restoration after the complete eruption of all the permanent teeth.

## CONCLUSION

Although the endodontics and prefabricated post and core build up restoration is an established procedure in dentistry, the prognosis of the same needs to be assessed on the basis of long-term periodic recalls.
